# Classification of Motor Functions from Electroencephalogram (EEG) Signals Based on an Integrated Method Comprised of Common Spatial Pattern and Wavelet Transform Framework

**DOI:** 10.3390/s19224878

**Published:** 2019-11-08

**Authors:** Norashikin Yahya, Huwaida Musa, Zhong Yi Ong, Irraivan Elamvazuthi

**Affiliations:** 1 Centre for Intelligent Signal and Imaging Research (CISIR), Department of Electrical and Electronic Engineering, Universiti Teknologi PETRONAS, Seri Iskandar 32610, Malaysia; 2 PETRONAS Carigali Sdn Bhd, Kerteh 24300, Malaysia; huwaidamusa@gmail.com; 3 Department of Electrical and Electronic Engineering, Universiti Teknologi PETRONAS, Seri Iskandar 32610, Malaysia; ozy2103@gmail.com; 4 Smart Assistive and Rehabilitative Technology (SMART) Research Group, Department of Electrical and Electronic Engineering, Universiti Teknologi PETRONAS, Seri Iskandar 32610, Malaysia; irraivan_elamvazuthi@utp.edu.my

**Keywords:** grasp-and-lift, CSP, CWT, scalogram, multiresolution, eigendecomposition, generalized eigen value decomposition, matrix joint diagonalization, oriented energy

## Abstract

In this work, an algorithm for the classification of six motor functions from an electroencephalogram (EEG) signal that combines a common spatial pattern (CSP) filter and a continuous wavelet transform (CWT), is investigated. The EEG data comprise six grasp-and-lift events, which are used to investigate the potential of using EEG as input signals with brain computer interface devices for controlling prosthetic devices for upper limb movement. Selected EEG channels are the ones located over the motor cortex, C3, Cz and C4, as well as at the parietal region, P3, Pz and P4. In general, the proposed algorithm includes three main stages, band pass filtering, CSP filtering, and wavelet transform and training on GoogLeNet for feature extraction, feature learning and classification. The band pass filtering is performed to select the EEG signal in the band of 7 Hz to 30 Hz while eliminating artifacts related to eye blink, heartbeat and muscle movement. The CSP filtering is applied on two-class EEG signals that will result in maximizing the power difference between the two-class dataset. Since CSP is mathematically developed for two-class events, the extension to the multiclass paradigm is achieved by using the approach of one class versus all other classes. Subsequently, continuous wavelet transform is used to convert the band pass and CSP filtered signals from selected electrodes to scalograms which are then converted to images in grayscale format. The three scalograms from the motor cortex regions and the parietal region are then combined to form two sets of RGB images. Next, these RGB images become the input to GoogLeNet for classification of the motor EEG signals. The performance of the proposed classification algorithm is evaluated in terms of precision, sensitivity, specificity, accuracy with average values of 94.8%, 93.5%, 94.7%, 94.1%, respectively, and average area under the receiver operating characteristic (ROC) curve equal to 0.985. These results indicate a good performance of the proposed algorithm in classifying grasp-and-lift events from EEG signals.

## 1. Introduction

The human brain is the central control system in the body, responsible for physical activities, processing, as well as interpreting the information received from sensory organs [1,2]. Understanding the human brain’s cognitive behavior is an appealing area for medical researchers in finding solutions for various cases with brain related disorders [3]. Brain computer interfaces (BCIs) based on electroencephalograms (EEGs), are one of the most clinically studied BCI technologies due to being a non-invasive technique, and having good temporal resolution, ease of use, portability, and a low set-up cost. There are significant research efforts using EEG-based BCI to restore limb function in patients suffering neurological damage either from accident or disease. The main idea is to have a control algorithm which processes EEG signals according to the user’s intention. However, the highly non-stationary and large intersession variation of EEG signals pose a great challenge in the development of EEG-based BCI.

Generally, EEG signals can be differentiated based on their amplitude, frequency and the position of the electrodes. Actions and behavior are most likely linked to β waves, as signals in this band are associated with the senses such as touch, hear, smell and taste. β waves, which cover the frequency range of 13 Hz to 30 Hz, occur in a conscious state, whereas μ waves appear in the range of 8 Hz to 13 Hz, associated with motor cortex functions [3]. These types of waveforms are extracted from electrodes located over the motor cortex, which are the C3, Cz and C4 locations that are responsible for body sensory and motor functions [1,3]. In addition, electrodes located at the parietal locations, such as P3, Pz and P4, can also be used to classify the motor movement signal as they are related to cognitive processing in the brain [4].

Raw EEG recordings may contain artifacts coming from external and internal sources. The main source of external artifacts is the 60 Hz power line frequency component [1] whereas the internal artifacts are due to bio electrical potentials from eye blinking or muscle potentials caused by the jaw and facial movements. According to Singhal et al. [5], the specific frequency related to artifact signals such as eye blinking is below 4 Hz, artifacts from heartbeats are around 1.2 Hz, while the frequency of muscle artifacts is above 30 Hz. One of the techniques to remove these artifacts is by applying a band pass filter to remove the unwanted frequencies while retaining the frequency band of interest. In addition, the EEG signal has its own drawbacks such as having a low signal-to-noise ratio and a low spatial resolution [6]. Therefore, researchers are finding better ways to extract features of synchronization and desynchronization events during motor imagery, which is achieved by using the common spatial pattern (CSP) method [7]. In addition, frequency domain analysis of a 1D signal can be efficient, especially for EEG signals that are sensitive to noise [8]. Wavelet transform converts 1D signals in the time domain to the 2D frequency domain and it can be analyzed at multiresolution. The 2D frequency domain signal is known as scalogram.

## 2. Related Work

During the past decade, the decoding of hand movements from EEG signals for assistive technology applications such as controlling a prosthetic device has been investigated. The research includes studies on decoding the movement of wrists [9,10,11], upper limbs [12], and elbows and shoulders [13]. In [11], Li et al. used connectivity analysis based on EEG as the feature for decoding voluntary right hand movement. It is established that the most sensitive EEG components are from the delta, theta, and gamma band of electrodes in the premotor and somatosensory motor cortex. In another work by Úbeda [12], assessment on the feasibility of decoding upper limb kinematics from EEG signals in center-out reaching task movements was investigated. Their results showed that arm movement decoding was significantly above the level that would be expected by random choices. The study also indicated that EEG signals below 2 Hz carry significant information to decode active center-out movements.

The common spatial pattern (CSP) feature extraction was initially applied in the classification of motor imagery EEG [14,15,16,17,18,19,20,21]. The CSP-based algorithms can effectively discriminate two different events by minimizing the variance of one class while maximizing the variance of another class, giving better classification accuracy [17,22]. Among the advantages of CSP are computational efficiency, ease of implementation on EEG data, and flexibility, as it can work for both synchronization and desynchronization events of BCI. In most cases, CSP-based methods are limited to two-class classification of EEG data since CSP requires simultaneous diagonalization [23]. However, in the case of classification for more than two classes, approaches such as one-versus-rest, divide and conquer, and joint approximate diagonalization can be used for multiclass extension [1,18,23].

In recent years, there have been many published works on the classification of motor imagery EEG signals based on CSP. Wang et al. in [7], proposed the selection of channels based on the maximum spatial pattern vectors in scalp mapping, combined with event-related desynchronization and readiness potential for classification of imagery hand and foot movement. In [23,24,25,26], methods that combined both filter bank and CSP are used for multiclass classification of motor imagery EEG signal. Using a filter bank, EEG data is separated into multiple frequency bands prior to CSP feature extraction and this has been shown to improve the classification accuracy of motor imagery tasks. In [26], Park et al. proposed subband regularized CSP for classification of motor imagery EEG, which used a filter bank that splits EEG signals in the range of 4–40 Hz into subbands of 4 Hz wide. The proposed method yielded classification accuracy with the range of 63.78% to 98.21% for five subjects.

Application of CSP for actual motor EEG signals was investigated by Robinson et al. [27]. The study aimed to decode directional information from the brain data collected during an actual hand movement experiment. Their proposed method utilized wavelet-common spatial pattern (W-CSP) algorithm to improve its direction-decoding accuracy. The W-CSP method applied a wavelet transform prior to CSP filtering for feature extraction. Subsequently, using a Fisher linear discriminant classifier, the recorded average accuracy range between 68.7% to 79.58% which was evaluated for seven subjects. In another work, Shiratori et al. [23], proposed a classification algorithm of actual three-class finger tapping task EEG data using filter bank and multiclass CSP which has resulted in accuracy of 88.7%.

In [28], Várszegi compared the performance of three algorithms for classification of EEG grasp-and-lift motor functions which resulted in an average area under the receiver operating characteristic (AUROC) between 0.722 to 0.829. Specifically, the EEG data of the first algorithm was passed through four processing stages of artifact rejection, band-pass filter, CSP filter, variance normalization and input to logistic regression ensemble for classification. This method resulted in an average AUROC of 0.722. In the second algorithm, the raw EEG was subjected to three processing stages of artifact rejection, low-pass filter band and normalization. Using a logistic regression classifier, the AUROC for the second algorithm was 0.796. The third algorithm applied artifact rejection and normalization to the EEG data and input to the 1D convolutional neural network (CNN) based on [29] for classification. The average AUROC for the third algorithm was 0.829, which is 4.1% and 14.8% better than the first and second algorithm, respectively.

In another work by Singhal et al. [5], an EEG recording of six-stage grasp-and-lift tasks was classified using CNN and a Butterworth low-pass filter. A total of 10 cascaded third order Butterworth low-pass filters were utilized on the FP1 electrode to filter out the artifact signal originating from eye blinking. The CNN used for their work has a five-layer structure, which include the data layer, 1D convolutional layer, max pooling layer and two fully connected layers. The data layer will connect the data set to the neural network. In the convolutional layer, 64 filters with size 1×3 were used, resulting in 64 output feature maps of size 1×62. A max-pooling layer, acting as a down sampling layer, was utilized to make the network more robust against feature offsets. Lastly, the two fully connected layers were used to connect and combine all the previous layers for calculation of the probability of the occurrence of six events in grasp-and-lift trials. The proposed architecture performs well with a final AUROC value of 0.91.

Combination of continuous wavelet transform (CWT) and CNN is often used in classification of time-series data [8,30,31,32]. This is taking advantage of the high frequency and time resolution provided by CWT, allowing detail analysis at various times and frequency. Here, the time-series is converted to the scalogram format prior to being input to the CNN. In [30], the downsampling method is used in the wavelet transformation where accuracy comparison between the deep neural network (DNN) and CNN has shown that max pooling and average pooling can provide better classification accuracy. In [31], a different approach was used for transforming acoustic data using the sliding window method into scalograms and spectrograms. Improvement in accuracy was obtained using transfer learning, achieved by transferring features learned in the fully connected layer of a pre-trained CNN into a general regression neural network (GRNN).

Based on the previous work on classification of motor EEG for BCI application, CSP is proven to be one of the major contributing factors for better classification of EEG data. In addition to CSP, the combination of CWT with CNN as the classifier, has proven to provide more discriminative features that result in good classification of EEG signals. Hence, in this work, we investigated the performance of a classification algorithm that uses CSP, CWT and GoogLeNet to classify grasp-and-lift motor function from EEG signals into six-class events. This paper contribution is focused on integration of a CSP–CWT framework with GoogLeNet, which will provide more comprehensive feature information related to a specific motor action. The decoding algorithm based on the CSP and CWT has a strong structure for data representation that facilitates precise classification. The successful classification of motor functions using EEG could facilitate the development of noninvasive BCIs with more controls and complicated movement functions.

This paper is organized as follows. Section 3 covers the methodology for classification of grasp-and-lift motor functions from EEG signals. Section 4 provides detail of the proposed CSP-CWT classification algorithm. Section 5, covers the experimental results and finally, Section 6 concludes the paper and suggests further work in this area.

## 3. Methodology

**Figure 1:**
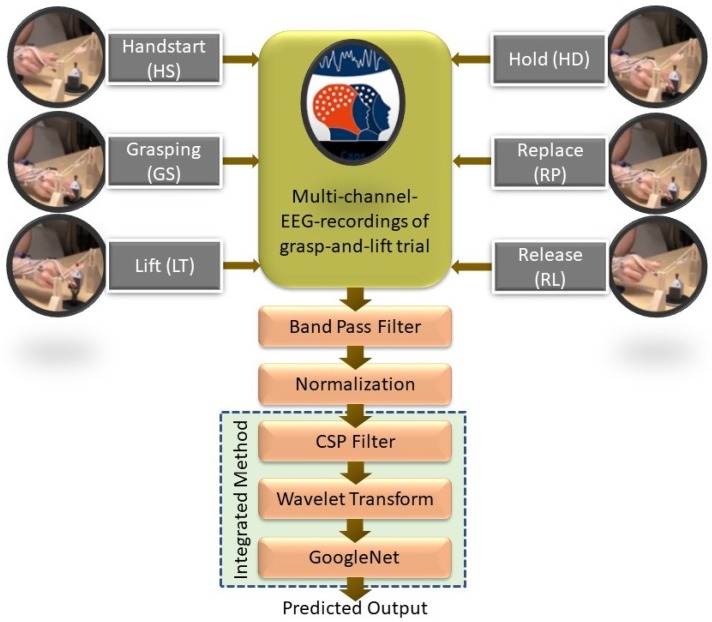
General framework for classification of motor electroencephalogram (EEG) events using a common spatial pattern (CSP) filter and a continuous wavelet transform (CWT).

The implementation of signal processing and a CSP–CWT framework with GoogLeNet for classification of grasp-and-lift EEG data is illustrated in Figure 1. EEG data collection for six grasp-and-lift events was made by using an EEG recording device, actiCAP, recording signals from 32 EEG sensors/electrodes. The EEG data from selected electrodes is band pass filtered to remove noise and normalized using mean value and standard deviation. Then, the signal is fed to a CSP filter to better discriminate the signal between classes before conversion to frequency domain using the CWT producing scalogram images. The feature learning from scalograms of different classes and their classification is performed by the pre-trained CNN network, GoogLeNet. Performance evaluation of the proposed algorithm is expressed in terms of classification accuracy, precision, sensitivity, specificity and AUROC.

### 3.1. Grasp-and-Lift (GAL) EEG Dataset

The grasp-and-lift EEG dataset was sponsored by the WAY consortium (wearable interfaces for hand function recovery) with the aim to understand the relationship between EEG signals, human movement, and BCI devices to aid patients suffering from neurological disabilities. Data collection was made using an EEG recording device, known as ActiCap that consists of 32 electrodes at a sampling frequency of 500 Hz.

**Figure 2:**
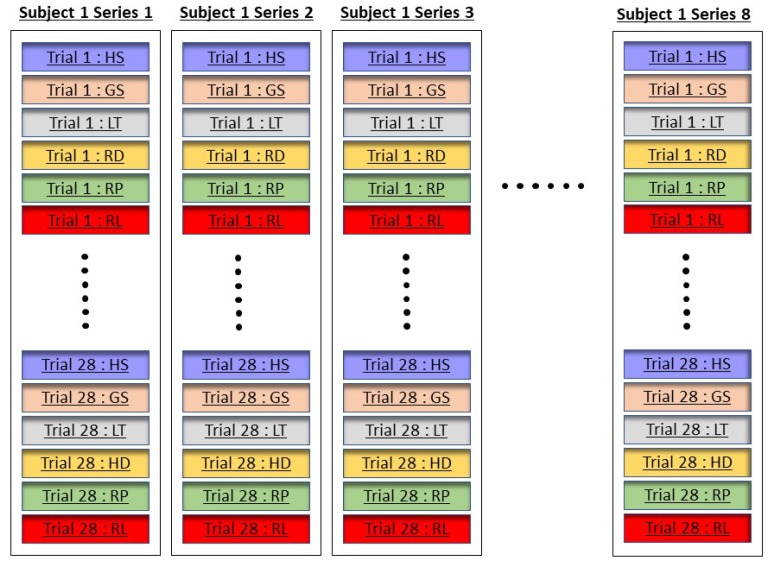
Arrangement of six grasp-and-lift events in EEG data collection for a single subject conducted for eight different series.

The dataset consists of EEG recordings of grasp-and-lift tasks collected from 12 healthy subjects where each subject performed 10 series of trials and there are approximately 30 trials within each series, as illustrated in Figure 2. The electrodes location was determined based on the 10/20 system positioning [33]. During data collection, each subject was instructed to reach their hand towards an object, lift it with their thumbs, hold it in the air, followed by placing it in different positions. The detailed descriptions and data label of six grasp-and-lift tasks are shown in Table 1. Complete description of the grasp-and-lift experiment is available in [34].

Event label of the dataset is captured by using the information from four 3D position sensors recording the position and orientation of the object, the index finger, the thumb and the wrist. The position is recorded in x, y and z Cartesian coordinates and the orientation is recorded in terms of azimuth, elevation, and roll. On the sides of the object there were two surface contact plates each coupled to a force transducer that recorded three force and three torque channels. To identify many events a combination of first and second time derivatives of the pertinent signals were employed. Before computing these derivatives, all signals were subjected to Savitzky–Golay filtering for the purpose of smoothing the data, that is, to increase the precision of the data without distorting the signal [34].

### 3.2. Channel Selection

Selection of EEG channels for the grasp-and-lift tasks will be the ones that carry most of the information related to the motor function. A total of six out of 32 channels are selected namely, C3, Cz, C4, P3, Pz, and P4 based on the correlation of the movement for the grasp-and-lift tasks [35]. The three channels C3, Cz and C4 are associated with motor movement, while P3, Pz and P4 are related to cognitive processing in the brain.

### 3.3. Band Pass Filtering and Data Normalization

Prior to CSP filtering, the EEG data is band pass filtered with cut-off frequencies of 7 Hz and 30 Hz to extract EEG signals in the μ and β band as shown in Figure 1. Selection of this band will eliminate the artifact related to eye blink (below 4 Hz), heartbeat (around 1.2 Hz), and muscle movement (above 30 Hz) in the EEG signals as these artifacts are basically outside the μ and β band. Band pass filtered EEG data are normalized by subtracting the mean and dividing by the standard deviation of the signal.

### 3.4. Common Spatial Pattern (CSP) Filtering

The CSP filtering is based on amatrix decomposition method that maximizes the power difference of the two-class signal. This is achieved by maximizing the variance in one class while minimizing the variance of the other class. In essence, CSP filtering will improve the discriminative power of EEG signals and result in better classification. Details on principle of CSP filtering are covered in Section 4.1.

### 3.5. Continuous Wavelet Transform

Continuous wavelet transform (CWT) is one of the commonly used methods for transforming 1D signals into a 2D matrix in frequency domain. The wavelet transform is known to be a more powerful method than the conventional cosine and Fourier transforms, as a time-frequency transform [36]. Comparing to Fourier transform which produces spectrograms with fixed time and frequency resolution, wavelet transform incorporates multiple scales and for this reason gives optimal time-frequency resolution [37]. The wavelet transform of CSP filtered EEG will produce a 2D matrix, known as a scalogram which is the absolute value of the CWT coefficients of a signal. The wavelet filter bank uses the analytic Morse wavelet with the symmetry parameter and time-bandwidth product equal to 3 and 60, respectively. The wavelet minimum and maximum scales are determined automatically based on the energy spread of the wavelet in frequency and time domain. Since the scalogram images of wavelet transform is 69×400, these images have to be resized to 224×224 using bicubic interpolation. This step is necessary because the pretrained GoogLeNet will only take input with an image size of 224×224.

### 3.6. GoogLeNet

Since the 1D EEG signal is converted to a spectrogram, this makes signal analysis morphologically complex. This means traditional machine learning techniques with handcrafted feature extraction will result in poor performance, hence CNN deep learning (GoogLeNet) is considered for this work. GoogLeNet (2015) [38] or Inception V1 CNN architecture was the winner of the ImageNet Large Scale Visual Recognition Challenge (ILSVRC) 2014 competition. GoogLeNet was built based on a design approach whereby the computational budget is kept constant when the network depth is increasing. This architecture has 22 layers consisting of the stem network, inception module connected in cascade, and classifier. The main contribution of this architecture is the utilization of a simple global average pooling across the 2D feature map after the convolutional layer to reduce the sum of parameters yet producing high accuracy results. In the work, a pre-trained GoogLeNet that takes scalogram images as input is utilized for classifying a six-class motor event from an EEG recording. GoogLeNet is considered a good choice of deep learning network for this work because it is one of the faster networks with relatively good accuracy and a small size for the network on disk.

### 3.7. Evaluation Metrics for the Proposed Algorithm

The performance evaluation of the proposed motor EEG events classification algorithm is based on the following measures as detailed in Equations (1)–(5).

(1) $$\mathrm{Precison}=\frac{\mathrm{TP}}{\mathrm{TP}+\mathrm{FP}}$$

(2) $$\mathrm{Sensitivity}=\frac{\mathrm{TP}}{\mathrm{TP}+\mathrm{FN}}$$

(3) $$\mathrm{Specificity}=\frac{\mathrm{TN}}{\mathrm{TN}+\mathrm{FP}}$$

(4) $$\mathrm{Accuracy}=\frac{\mathrm{TP}+\mathrm{TN}}{\mathrm{TP}+\mathrm{TN}+\mathrm{FP}+\mathrm{FN}}$$

where TP: True Positive, TN: True Negative, FP: False Positive, FN: False Negative. Apart from the above performance metrics, the area under the ROC curve will also be used to evaluate the performance of the proposed algorithm. The receiver operating characteristic (ROC) curve is a plot of true positive rate against the false positive rate (1-specificity) at different classification threshold settings which gives a measure of diagnostic ability of a two-class classifier. In addition, ROC analysis also allows analyzing sensitivity and specificity simultaneously at different cut-points and hence gives a better estimate on the accuracy of a given trial test by using multiple pairs of sensitivity and specificity. The true positive rate is also known as sensitivity and is shown in Equation (2) and the false positive rate is defined as follows,

(5) $$\mathrm{False}\mathrm{positive}\mathrm{rate}=\frac{\mathrm{FP}}{\mathrm{FP}+\mathrm{TN}}.$$

## 4. Proposed Model

During the pre-processing stage, EEG signals in the frequency band of 7 Hz to 30 Hz are selected as this band contained the μ and β band. The μ band with a frequency range of 8–12 Hz is related to movement activity associated with the motor cortex, whereas the β band with a frequency range of 16–30 Hz are the ones that occurred during conscious states such as talking, problem solving and decision making. Therefore selection of EEG signals in frequency bands between 7 Hz to 30 Hz will capture important signals related to motor functions.

**Figure 3:**
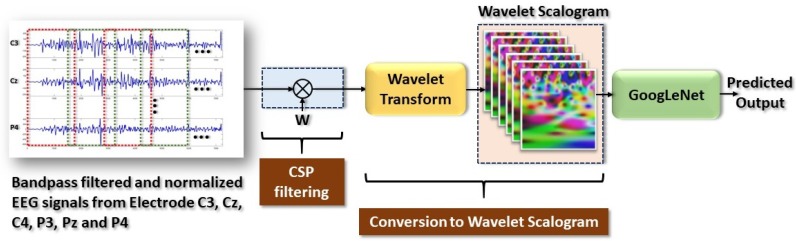
General framework for classification of EEG motor function data using CSP and CWT.

As such, a band pass filter with a pass band frequency of 7 Hz to 30 Hz is used to capture the desired band in the pre-processing stage. A common spatial pattern (CSP) filter is utilized to extract the common spatial features from the EEG signal that has the maximum variance between two classes. Since the technique used a two-class CSP filter, the one-versus-rest scheme is used for every class. Data is separated into event and non-event, and then multiplied with a spatial filter, *W*, which is determined using Equation (12). The sliding window method with window size of 400 samples and overlap of 100 samples is used to form grayscale scalograms from each electrode. Figure 3 illustrates the procedure of preprocessing, CSP feature extraction, and conversion to wavelet scalograms.

**Figure 4:**
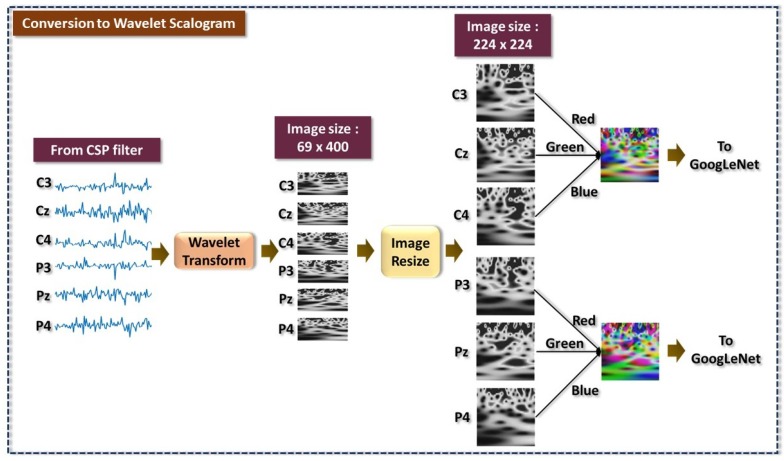
Formation of scalogram images of EEG motor function from electrode C3, Cz, C4, P3, Pz and P4 using CWT.

An illustration of scalogram conversion, image resizing and formation of RGB images as input to GoogLeNet is shown in Figure 4. Every scalogram is labelled according to their respective class based on the one-versus-rest scheme. Specifically, there are six groups of grayscale scalograms corresponding to the six selected electrodes including C3, Cz, C4, P3, Pz and P4. With a 400-sample EEG, the CWT output scalogram has a dimension of 69×400. It is known that GoogLeNet will take in input of size 224×224, hence, the 69×400 scalogram images need to be resized and this is achieved using cubic interpolation. Subsequently, the 224×224 scalograms from electrodes C3, CZ, and C4 are concatenated to form a set of RGB scalograms. The same procedure is applied on data from electrodes P3, Pz, and P4.

### 4.1. Common Spatial Pattern

Common spatial pattern (CSP) is a time-series feature extraction method mostly used in the BCI field for classification of two-class event [6,7,14,39] using imagery EEG data. CSP is able to extract spatial features of two events and able to maximize the power difference on the resulting outputs of different EEG classes. In achieving maximum power difference, the variance between the two classes is maximized and doing so will maximize variance in one class while minimizing variance of the other class. Details of the CSP algorithm are as follows. Let $X_{1}$ and $X_{2}$ denote the two different classes of EEG grasp-and-lift events with dimension equal to N×T, where *N* is the number of channels and *T* is the number of samples per channel. Equations (6) and (7) below are the normalized spatial covariance, $R_{1}$ and $R_{2}$ of EEG for class 1 and class 2, respectively.

(6) $$R_{1}=\frac{X_{1}X_{1}^{T}}{trace(X_{1}X_{1}^{T})}$$

(7) $$R_{2}=\frac{X_{2}X_{2}^{T}}{trace(X_{2}X_{2}^{T})}.$$

Here, $X^{T}$ denotes the transpose of *X* and trace(X) is the summation of diagonal elements in *X*. The average value of normalized covariance, ${\bar{R}}_{1}$ and ${\bar{R}}_{2}$ are computed by finding the average of all trials for each class. Thus, the eigenvalue decomposition of composite spatial covariance is expressed as

(8) $$R_{12}={\bar{R}}_{1}+{\bar{R}}_{2}=U\Delta U^{T},$$

where *U* and Δ mark the eigenvectors and diagonal matrix of the eigenvalues, respectively. The eigenvalues are then organized in descending order before being used to obtain the whitening transformation matrix, $P_{w}$,

(9) $$P_{w}=\Delta^{\frac{-1}{2}}U^{T}$$

to decorrelate the normalizing spatial covariances and giving a transformed average covariance matrix equal to

(10) $$Q_{1}=P_{w}R_{1}P_{w}^{T}\;\mathrm{and}\;Q_{2}=P_{w}R_{2}P_{w}^{T}.$$

$Q_{1}$ and $Q_{2}$ share similar common eigenvectors and the result for the summation of eigenvalues of the two matrices is equal to one,

(11) $$Q_{1}=V\Delta_{1}R_{1}V^{T}\;\mathrm{and}\;Q_{2}=V\Delta_{2}R_{2}V^{T},\;\Delta_{1}+\Delta_{2}=I.$$

Here, *V* is the common eigenvectors, and $\Delta_{1}$ and $\Delta_{2}$ are eigenvalues of $Q_{1}$ and $Q_{2}$, respectively. The addition of these eigenvalues gives the largest eigenvalues of $Q_{1}$ to be the smallest eigenvalues of $Q_{2}$ and the other way around. Essentially, the whitened EEG approached is optimal for the purpose of separating the variance of two signal matrices, $R_{1}$ and $R_{2}$ [14,40]. This will improve the classification of EEG since selected channels can better distinguish the two classes. Then, the spatial filter, *W* is computed as

(12) $$W=V^{T}P_{w}.$$

From Equation (12), the original EEG data is transformed to uncorrelated components, *Z* by using the projection matrix, *W*,

(13) Z=WX.

From here, the original EEG data can be restored by performing the following operation,

(14) $$X=W^{-1}Z$$

where $W^{-1}$ is the inverse matrix of *W*. Notably, the columns of $W^{-1}$ are the CSP which also represent the time-invariant EEG source. The first and last columns of $W^{-1}$ are noted as the spatial patterns that contain the largest variance in one class and the smallest variance in the other class.

**Figure 5:**
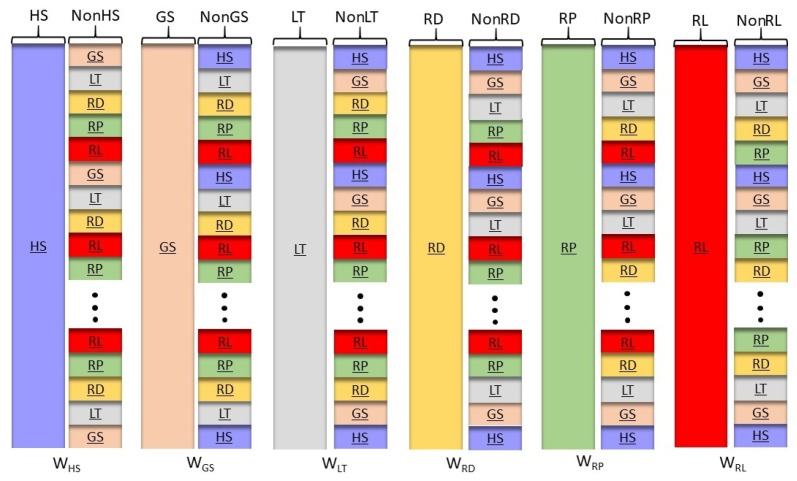
One-versus-rest arrangement of EEG data for calculation of CSP spatial filters of six motor events, $W_{HS},W_{GS},W_{LT},W_{HD},W_{RP}$ and $W_{RL}$.

Computation of the spatial filters, for the six-class event, $W_{HS},W_{GS},W_{LT},W_{HD},W_{RP}$ and $W_{RL}$, will be using the one-vs-rest method. In this case, the data from the six events will be arranged in the form of handstart (HS) vs. NonHS, grasping (GS) vs. NonGS, lift (LT) vs. NonLT, hold (HD) vs. NonHD, eplace (RP) vs. NonRP, and release (RL) vs. NonRL as shown in Figure 5. Note that for the non-event (NonHS, NonGS, etc), there are 120 possible unique combinations of five events to be included in the non-event matrix.

The CSP feature for each one-vs-rest dataset will be transformed to 2D scalograms using continuous wavelet transform. In essence, the conversion of CSP to wavelet scalogram offers the possibility of hierarchical feature learning by the GoogLeNet which captures both high-level semantics and low-level spatial details. The integration of the CSP–CWT framework with GoogLeNet will play a critical role for better classification of hand movement EEG signals.

## 5. Results

This section begins with a brief analysis on the grasp-and-lift EEG recordings, the band pass filtered data, and the CSP filtered data. Then, the experimental results on the classification of the six-class motor events using CSP, CWT and pre-trained GoogLeNet, abbreviated as the CSP–CWT method, is investigated. A comparison with two other algorithms that used the same dataset is also conducted. Training and testing of GoogLeNet was implemented on the Matlab platform and ran on a laptop equipped with 16 GB memory, an Intel i7-8750H CPU, and a Nvidia GTX 1060 graphic card with 6 GB video memory.

### 5.1. Band Pass Filtering of EEG Signals

**Figure 6:**
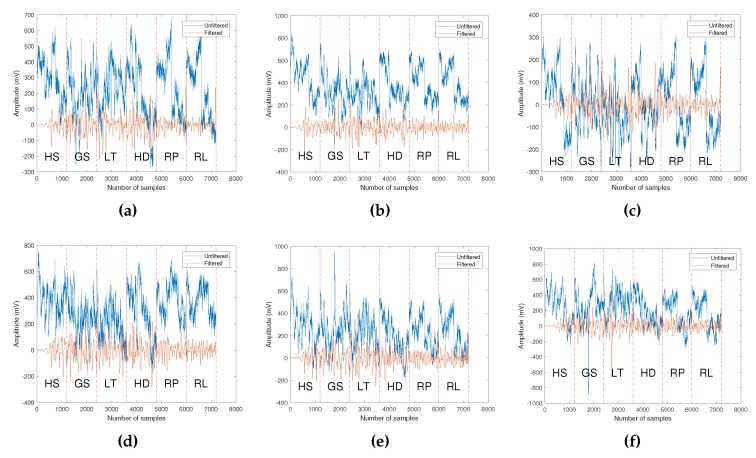
The 14.4 s duration of the original and band pass filtered EEG recordings for subject 1 from electrode (**a**) C3, (**b**) Cz, (**c**) C4, (**d**) P3, (**e**) Pz, and (**f**) P4, comprising the six grasp-and-lift motor events, HS, GS, LT, HD, RP and RL.

The raw and band pass filtered EEG data comprised of six grasp-and-lift events for subject 1 from the six channels are as shown in Figure 6. The band pass filtered signal will retain a signal between a frequency of 7 to 30 Hz, and this eliminates artifacts such as eye blinking, power line noise, muscle artifacts and heartbeat artifacts. Using an equiripple finite impulse response (FIR) filter, the filter lower stopband frequency is set at 5 Hz whereas the upper stopband frequency is at 33 Hz. The attenuation of the stopbands is set at 65 dB.

For each trial, the grasp-and-lift tasks are performed in sequential order of HS, GS, LT, HD, RP and RL as shown in Figure 6. From Figure 6, it can be seen that the EEG signal from six electrodes have different amplitude range, but the signal trend is not clearly visible. Hence, CWT that transforms the signal to time-frequency domain will be used for classification of grasp-and-lift tasks by a pre-trained CNN.

### 5.2. Common Spatial Pattern (CSP) Filtering

**Figure 7:**
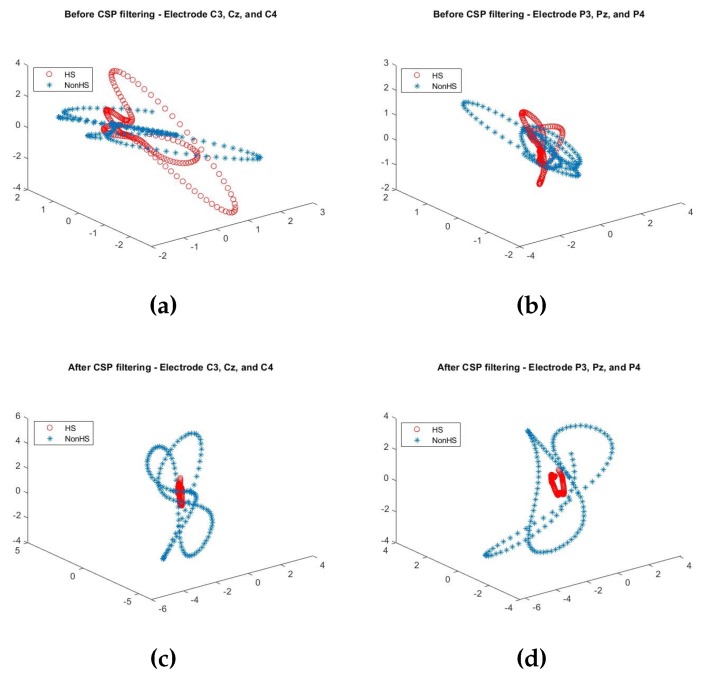
The 3D-scatter plots of 300 samples of EEG for HS vs. Non-HS events from (C3, Cz, C4) electrodes and (P3, Pz, P4) electrodes; where (**a**,**b**) are the plots before CSP filtering, and (**c**,**d**) are the plots after CSP filtering.

As discussed in Section 4.1, in the data transformed by CSP filtering, *W* will result in largest variance in one class with the smallest in the other class. This can clearly be seen from the 3D-scatter plot of the (C3, Cz, C4) electrodes and (P3, Pz, P4) electrodes, shown in Figure 7, generated for HS vs. NonHS events. Notably, before CSP filtering, HS values are scattered over a larger 3D space but after CSP filtering, the values are mapped to a much smaller 3D space. Similar trend can be observed for the other five events.

**Figure 8:**
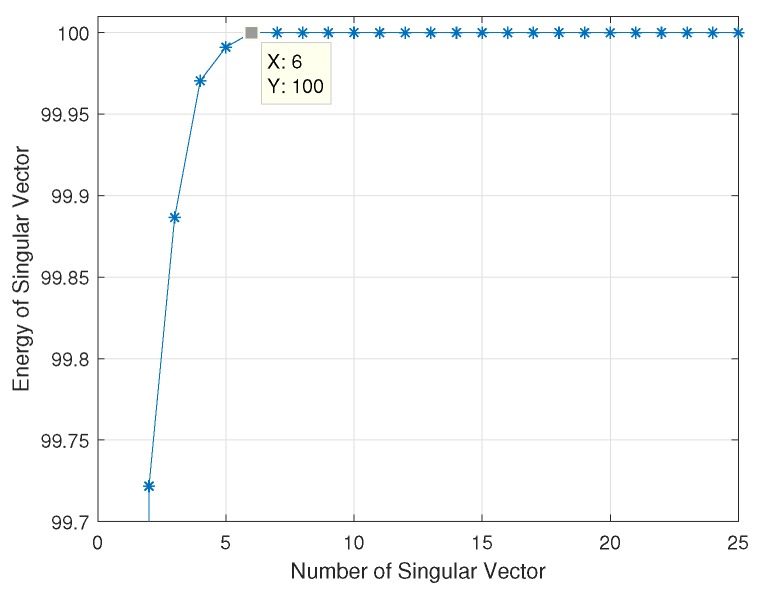
Energy measured in the subspace of the first 100 singular vectors of transformed EEG data, *Z*.

Another important property of CSP features which make it work so well in two-class event classification is the concept of maximal oriented energy. Since CSP is a subspace-based technique, most of the feature energy is compressed in the first few singular vectors of the CSP feature matrix, *Z* [41,42,43,44]. Figure 8 shows the plot of the energy content in the first 25 singular vectors of matrix *Z*. The graph shows that most of the feature energy is compressed in the first six singular vectors of CSP feature matrix *Z*. This is because the six eigenvectors are the vectors that defined the principal subspace that senses a maximal oriented energy of the covariance matrix of the six-channel EEG signals [45,46,47].

### 5.3. Activation of GoogLeNet Convolution Layer

**Figure 9:**
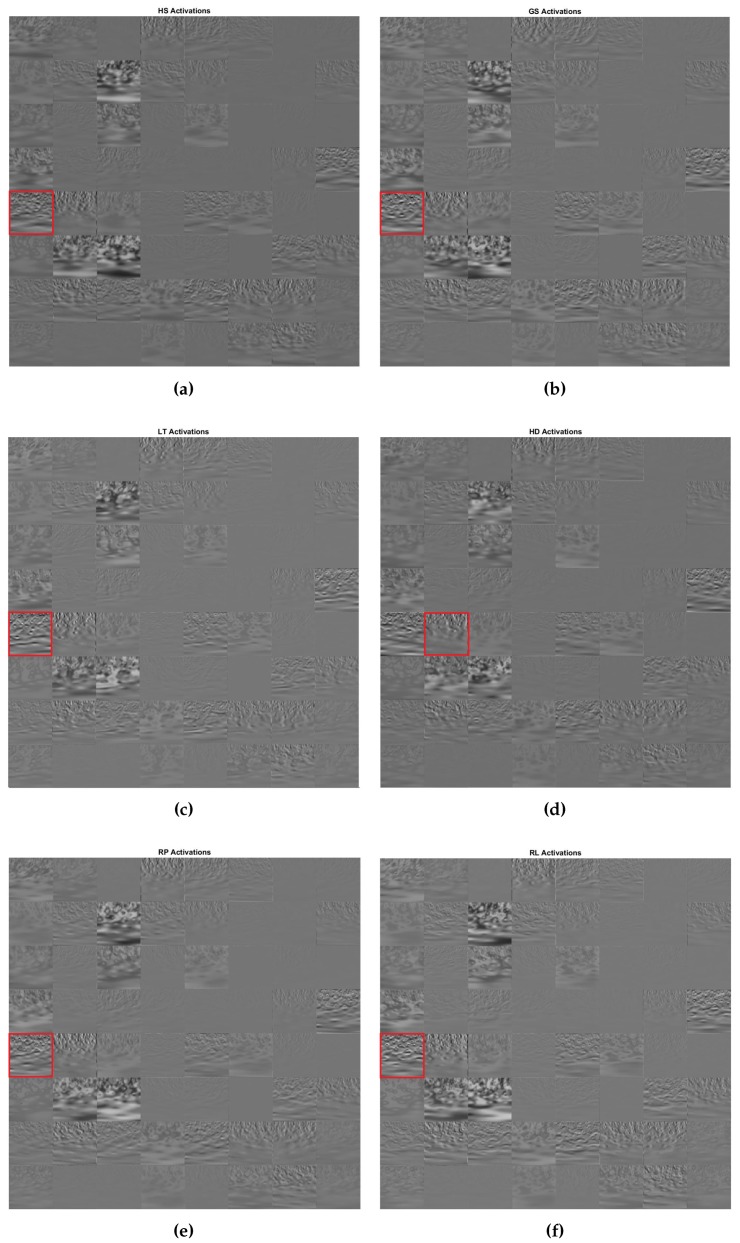
Output activation of the GoogLeNet first convolutional layer, conv1-7×7 for six motor events, (**a**) HS, (**b**) GS, (**c**) LT, (**d**) HD, (**e**) RP, and (**f**) RL tested in the framework of one-versus-rest. The square red box indicates the filter with the strongest activation.

Figure 9 shows the activation of the first convolution layer of GoogLeNet when fed with one image from each class of the six motor events. The channel with the strongest activation is marked by a square red box. Channel 33 shows strong activation for class HS, GS, LT, RP and RL, whereas Channel 34 shows strong activation for event HD. Visualization of how an image activates convolutional layers in the subsequent convolutional layers will give insight of what the network has learned from the input images.

### 5.4. Classification of Motor Events using CSP, Continuous Wavelet Transform and GoogLeNet

In this experiment, we evaluate the performance of the CSP–CWT algorithm for classification of EEG grasp-and-lift motor functions using GoogLeNet. It is important to highlight that data from 12 subjects are combined to generate a six-class dataset of equal length with the assumption that there are minimal intra-session and inter-session variations for the same task. Hence, to consider conditions that are close to a real BCI scenario, training of GoogLeNet should consider two frameworks, (1) leave-one-trial-out for subject dependent and (2) leave-one-subject out for subject-independent. The trained network should then be tested with the leave-one subject or leave-one trials.

Here, the role of feature extraction and classifier is taken by the deep learning network, GoogLeNet which is capable of capturing both high-level features and low-level spatial feature of scalograms. The size of the sliding window used for conversion to wavelet scalogram is at 400 samples, equivalent to 0.8 s duration with 100 samples overlapped. Each electrode will produce a scalogram in grayscale format. Three grayscale scalograms of size 224×224×1 from electrode C3, Cz and C4 of the same group are concatenated to form a RGB scalogram of size 224×224×3. Similar RGB scalograms will be generated from electrode P3, Pz and P4. In total, there are 5382 scalograms generated for every class of HS, GS, LT, HD, RP and RL.

**Figure 10:**
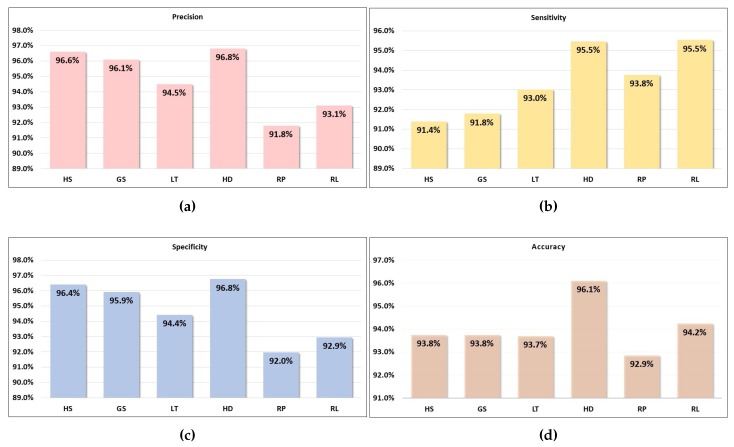
Performance evaluation for classification of six motor events tested in the framework of one-versus-rest based on percentage (**a**) precision, (**b**) sensitivity, (**c**) specificity, and (**d**) accuracy.

The RGB scalograms are separated into training image, validating image and testing image with the ratio of 0.8:0.1:0.1, respectively. Pre-trained GoogLeNet is utilized to train the model for classifying the six classes. Validating data are used to validate the accuracy in every epoch to prevent overfitting whereas testing data was used for accuracy testing after the training process. Testing scalograms of each class are fed into their respective trained model to validate the testing accuracy. Max epochs under the training option are set to be 30 epochs while mini batch size is set to be 10. The performance result for classification of six motor events by the CSP–CWT method, tested in the framework of one-versus-rest is presented in terms of precision, sensitivity, specificity and accuracy as shown in Figure 10.

From graph shown in Figure 10, we can see that the average value for precision, sensitivity, specificity and accuracy is at 94.8%, 93.5%, 94.7%, and 94.1%, respectively. These values show the good performance of the proposed method in classification of grasp-and-lift motor events from EEG signals. In terms of precision, the value for six classes varies between 91.8–96.7%. In particular, HD vs. NonHD has the highest precision whereas RP vs. NonRP has the lowest precision. Precision, which is also known as positive predictive value is the fraction of correctly classified motor events (one-versus-rest) among the retrieved instances. Sensitivity in this context is the probability that a grasp-and-lift motor event is correctly classified. It has range of values between 91.4% to 95.5%. The highest sensitivity value is recorded for two events, HD vs. NonHD and RL vs. NonRL. On the other hand, the HS vs. NonHS has the lowest sensitivity among the six grasp-and-lift events. On the other hand, specificity is the proportion of the NonEvent (NonHS, NonGS, NonLT, NonHD, NonRP and NonRL) instances correctly identified by the classifier and it has a range between 92.0% to 96.8%. The highest specificity value is by HD vs. NonHD and the lowest value is by RP vs. NonRP. Lastly, for the accuracy which measures the rate of correct classification of Event and NonEvent, the values ranged between 92.9% to 96.1% with the highest accuracy for HD vs. NonHD, and the lowest for RP vs. NonRP. Overall, it is clear that the algorithm performance is above average for the class of HD vs. NonHD.

**Figure 11:**
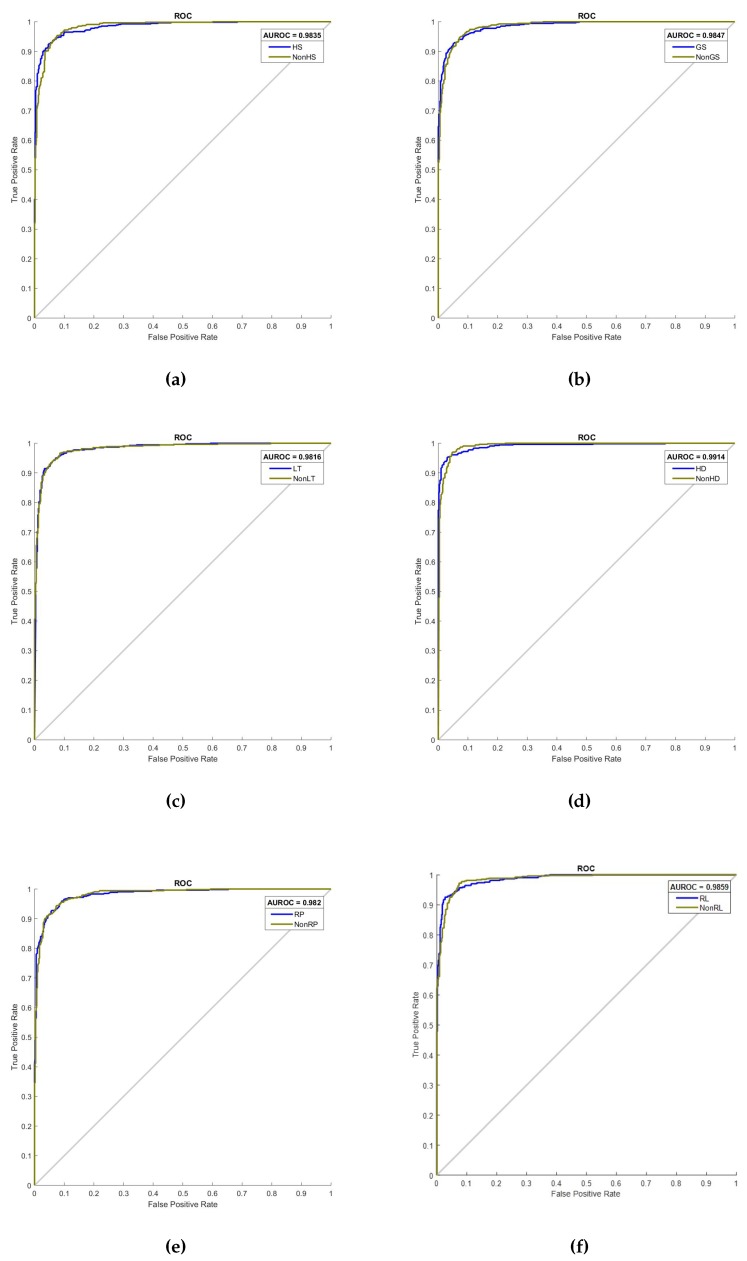
Receiver operating characteristic (ROC) curves for classification of six motor events, (**a**) HS, (**b**) GS, (**c**) LT, (**d**) HD, (**e**) RP, and (**f**) RL tested in the framework of one-versus-rest.

**Figure 12:**
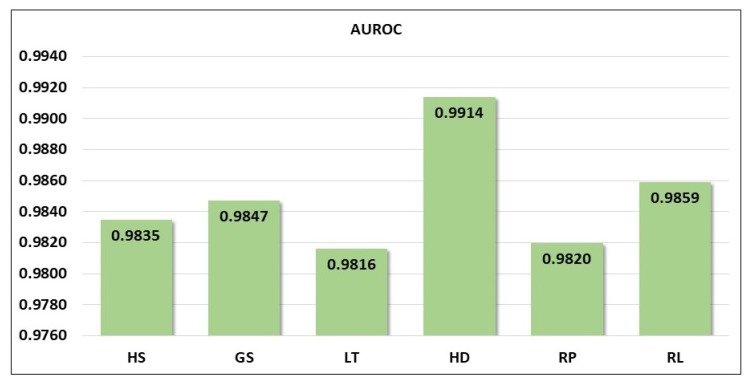
Area under the ROC for six motor events tested in the framework of one-versus-rest.

The plot of the ROC curve for the six motor events using the CSP–CWT method is given in Figure 11, shown with the value of area under the ROC (AUROC). The summary of AUROC values for six grasp-and-lift events are shown as a bar plot in Figure 12. The ROC curve allows simultaneous assessment of sensitivity and specificity at different cut-points which gives a better estimate on the accuracy of the classification algorithm. The plots show the range of the AUROC value is between 0.9816 to 0.9914 and the average value of AUROC is 0.9850. The two lowest AUROC values are recorded by LT vs. NonLT and RP vs. NonRP, whereas the highest value is by HD vs. NonHD. These results indicate that the HD event is the most discriminative event compared to the five other events, as it has the highest recorded values of AUROC, accuracy, specificity, sensitivity and precision.

For comparison, we tabulate the AUROC value of the CSP–CWT method with two other published methods as summarized in Table 2. From the result, the proposed CSP–CWT method has a recorded AUROC value that is 0.071% and 0.152% higher than the methods proposed in [5,28], respectively. The lower value of AUROC recorded in [5,28] is expected since the size of 1D CNN used for classification is a relatively small network compared to GoogLeNet. The slightly better performance of [5] is due to the introduced method of Butterworth low pass filters, before the CNN, to increase the number of channels from 32 to 52.

## 6. Conclusions and Further Work

We proposed an algorithm for classification of grasp-and-lift motor functions from EEG signals based on CSP and wavelet transform (CSP–CWT). The CSP has resulted in the EEG signals of different classes to be highly discriminative, which was achieved through CSP maximized variance between two classes, and resulted in a maximum power difference. Using generalized eigenvalue decomposition, CSP maximizes the variance in one class while minimizing the variance in the other class. In addition, it is also shown that CSP contains the six eigenvectors which is the principal subspace that senses a maximal oriented energy of the feature. This property is of great advantage to classifiers as this will ease the process to determine the separation boundary between any two classes. Wavelet transform offers the advantage of high frequency and time resolution, allowing detail analysis at all times and frequency. Using CWT, a 1D time-series EEG signal is converted to a 2D scalogram, which is used as the input to GoogLeNet. With an average accuracy of 94.1% and average AUROC of 0.985, this result has demonstrated the effectiveness of the CSP–CWT based method for classification of EEG grasp-and-lift motor functions.

This work was investigated with the assumption of minimal intra-session and inter-session variation, so the inherently high correlation of samples within individuals was not taken into consideration. In fact, to account for real BCI application, further experimentation using leave-one-trial-out scenarios or leave-one-subject out scenarios should be carried out. Besides, this issue will also affect the computation of the projection matrix, *W*, hence a regularization method, as proposed in [48,49], to overcome the sensitivity of CSP to outliers and overfitting should be employed. Future work should investigate the CSP–CWT algorithm with regularization in a more realistic BCI framework. 

## Figures and Tables

**Table 1:** Details of six-class grasp-and-lift tasks [34].

GAL Event	Description
Handstart (HS)	Reaching for the object
Grasping (GS)	Grasp the object using thumb and index finger
Lift (LT)	Lift force
Hold (HD)	Lifting an object for a couple of seconds
Replace (RP)	Place the object back on the support surface
Release (RL)	Release the object and place hand at the start position

**Table 2:** Performance comparison of classification of six motor events using the GAL dataset [34] based on the area under ROC (AUROC).

Method	Pre-Processing	AUROC
Proposed (CSP–CWT)	Preprocessing: CSP filtering and CWT CNN architecture: GoogLeNet 22-layer 2D CNN Evaluation: AUROC for every event from 12 subjects Number of Channel: six-channel (C3, Cz, C4, P3, Pz and P4)	0.985
Singhal, et al. (2019) [5]	Preprocessing: Butterworth Low Pass Filter CNN architecture: five-layer 1D CNN Evaluation: Average AUROC of 12 subjects Number of Channel: 32-channel	0.910
Várszegi (2016) [28]	Preprocessing: Artifact rejection and normalization CNN architecture: six-layer 1D CNN Evaluation: Average AUROC of 12 subjects Number of Channel: 32-channel	0.829
